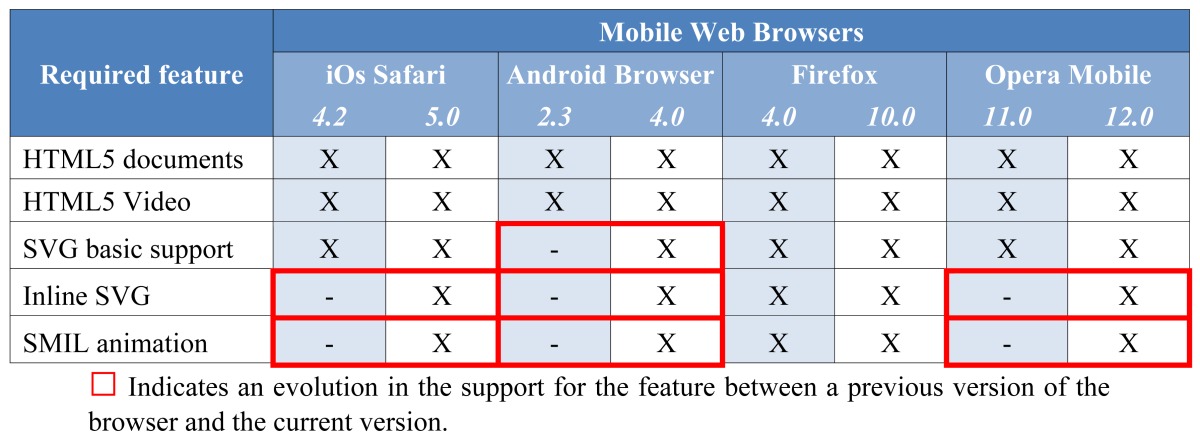# Subtitle Synchronization across Multiple Screens and Devices

**DOI:** 10.3390/s120708710

**Published:** 2012-06-26

**Authors:** Aitor Rodriguez-Alsina, Guillermo Talavera, Pilar Orero, Jordi Carrabina

**Affiliations:** Center of Ambient Intelligence and Accessibility of Catalonia, Universitat Autònoma de Barcelona, 08193 Bellaterra, Spain; E-Mails: Guillermo.Talavera@uab.cat (G.T.); Pilar.Orero@uab.cat (P.O.); Jordi.Carrabina@uab.cat (J.C.)

**Keywords:** Ambient Intelligence, accessibility, HTML5, subtitling, multimedia synchronization, SMIL

## Abstract

Ambient Intelligence is a new paradigm in which environments are sensitive and responsive to the presence of people. This is having an increasing importance in multimedia applications, which frequently rely on sensors to provide useful information to the user. In this context, multimedia applications must adapt and personalize both content and interfaces in order to reach acceptable levels of context-specific quality of service for the user, and enable the content to be available anywhere and at any time. The next step is to make content available to everybody in order to overcome the existing access barriers to content for users with specific needs, or else to adapt to different platforms, hence making content fully usable and accessible. Appropriate access to video content, for instance, is not always possible due to the technical limitations of traditional video packaging, transmission and presentation. This restricts the flexibility of subtitles and audio-descriptions to be adapted to different devices, contexts and users. New Web standards built around HTML5 enable more featured applications with better adaptation and personalization facilities, and thus would seem more suitable for accessible AmI environments. This work presents a video subtitling system that enables the customization, adaptation and synchronization of subtitles across different devices and multiple screens. The benefits of HTML5 applications for building the solution are analyzed along with their current platform support. Moreover, examples of the use of the application in three different cases are presented. Finally, the user experience of the solution is evaluated.

## 1. Introduction

**Figure 1.**
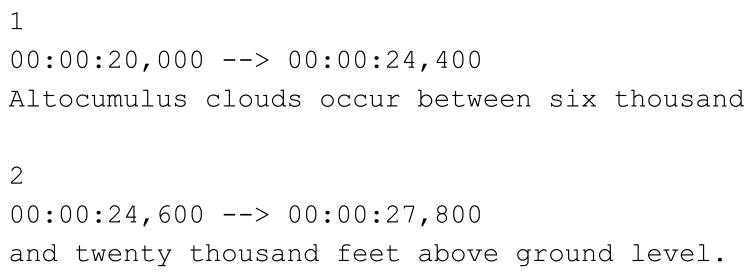
Example of subtitles defined using the SubRip (.SRT) format.

Multimedia applications are a key factor in Ambient Intelligence (AmI) environments in which products and services are responsive to the user context, offering a rich variety of applications in the professional and consumer domains. Both sensors included in the environment and devices can provide context information (connected devices, light conditions, noise levels *etc.*) and user information (profile, presence, movements *etc.*) in order to enhance the adaptability of the system to the needs of the user, thus enabling content to be available anywhere and at any time [1]. Nowadays, hearing and sight are the most natural ways of engaging in user interaction, and multimedia applications must also dynamically adapt to different users who may well have different sensorial capacities. Proper access to content within AmI environments also ensures that content is available to anybody. As with any multimedia context, in these environments, subtitles are one of the key elements for providing fast and efficient access to video content. Subtitles or captions, as a specific text type, offer a multifunctional service for breaking down the many barriers related to language and sound. The former are known as “interlingual subtitles”, which translate the language of the original version to the language of the target audience. The latter (labeled “intralingual subtitles”) are better known as subtitles or captions for the deaf and hard of hearing (SDHH). While in some cases, intralingual subtitles may also translate the language, SDHH usually render into written form that which is spoken on screen, together with all the sounds which are present in the audiovisual text, including music and lyrics. Their function goes beyond mere media accessibility and enters the realm of social integration, and of education, since they are powerful teaching tools for non-native speakers, for learning literacy and language, and for highlighting topics which are of particular relevance. Whilst there is general agreement as to the positive role of subtitles in language learning, their function is in fact far more beneficial than expected. Recent experiments [2] have shown how attention is heightened when subtitles are present; whilst there is an initial negative reaction to a double visual input and information overload, visual images and subtitles coexist, demanding a higher cognitive processing. Recent research [3] has shown how subtitles have further functions and are now entering the realms of language therapy and remedial education. Subtitle services enrich AmI environments by providing the appropriate complementary information at the appropriate time and in the appropriate place, depending on the user's needs, context and the devices available to the user in a specific environment. However, the mechanisms for subtitle presentation within AmI environments (as well as other accessibility services such as audio-description and sign language) do not yet conform to the accessibility requirements of specific user groups. At home, where users share and interact with a variety of smart devices (e.g., TVs, mobiles, tablets, PCs, laptops, consoles *etc.*), in order to access video content, the underlying technological differences between connected devices can be overcome through content adaptation techniques [4] and the use of web-based technologies. This enables the implementation of more ubiquitous systems via the widespread support of web applications across the variety of proprietary runtime environments provided by device manufacturers. However, most of the web video content which is consumed does not include selectable subtitles, and non-standard embedded video players do not allow for the independent management of subtitles with the aim of enabling adaptation to the accessibility requirements of the user (or application). Most subtitles distributed on the Internet are described in text files that follow the SubRip (.SRT) format, considered to be “perhaps the most basic of all subtitle formats” [5]. Figure 1 shows an example of a .SRT file. Each subtitle entry consists of the subtitle number, the time at which the subtitle should appear on screen, the subtitle itself, and a blank line to indicate the subtitle's end.

While the standardization of subtitles as part of HTML5 video and audio components is still being debated [6], novel approaches for video subtitling within web-based environments are required for the exploration of video accessibility within AmI environments. This article presents an alternative design strategy for implementing customizable subtitle systems for a variety of platforms and devices. It takes advantage of the native support for Scalable Vector Graphics (SVG) and the Synchronized Multimedia Integration Language (SMIL) [7] within HTML5 documents for presenting synchronized subtitles on any web-capable device with the appropriate browser. The current platform's support for the proposed solution is also analyzed, both for desktop environments and for those of mobile and TV. Furthermore, in order to demonstrate the viability and support for the proposed solution, three instances for the use of the application for video subtitling have been created: (1) a web widget to extend the functionality of an HTML5 video component, enabling user modifications to the language and format of the subtitles; (2) an add-on for smart TV platforms that improves subtitle readability when the user interface combines video with other interactive content; and (3) a mobile application to enable multi-screen capabilities within a home environment (as an example of an AmI environment) to receive TV subtitles on the mobile device regardless of the native runtime of the device.

The rest of the article is structured as follows: Section 2 analyzes the pros and cons of deploying Web applications compared to native applications; Section 3 presents an approach to synchronizing web-based video subtitles using SVG and SMIL; Section 4 analyzes the current platform support for the proposed approach; Section 5 presents examples of the use of the application in order to demonstrate the flexibility and viability of the solution; Section 6 describes the user experience evaluation conducted to validate the quality and acceptability of the application proposed as a solution for multi-screen systems; and finally Section 7 outlines the paper's conclusions and discusses the future.

## 2. Web Applications *vs.* Native Applications on Mobile Platforms

With the explosion in the popularity of smart devices and different marketplaces for applications, and the enhancement of Web technologies led by HTML5, it seems natural to analyze the pros and cons of building native applications *vs.* the development and maintenance of Web sites. This section compares the two, and justifies our choice for building the presented subtitling system for AmI environments, taking advantage of HTML5 capabilities.

### 2.1. Platform Support

Native applications need to be specifically built for every target platform. Thus, application providers must take into account the number and types of target platforms in order to address the application to each one's individual needs. It is not only a matter of the application environments (e.g., desktop, mobile, interactive TV *etc.*) or brands (e.g., iPhone, Android, BlackBerry *etc.*), but also a question of the platform version (Android, for example, has a large number of versions with different features depending on the device manufacturer). When the application needs to be supported by a new platform, it must be rewritten using its native programming language and its native functions relating to its look and feel.

In contrast, Web applications are based on Web standards and are deployed on a Web browser, which acts as a bond between an application written using shared standards and the specific runtime environment of each target platform. This enables Web applications to be deployed on any browser-capable platform, regardless of the application environment of the specific device. However, differences still exist when using the same platform with different browsers, due to the different implementation of features in the browser engine (for example, individual browsers support a different set of features for HTML5, and this may also depend on the browser version). Moreover, universal support for Web applications can be limited when using special device features such as the accelerometer for mobile devices and the tuner for TV platforms. Although HTML5 provides support for accessing some of these features (such as geolocation) through specific Application Programming Interfaces (APIs), it is quite clear that HTML5 will not support all the concrete features for all devices. When a Web application needs to be supported by a new platform, nothing more has to be done (at least theoretically, based on the principle of “build once, run anywhere”) than to ensure that the content fits properly within the new environment.

### 2.2. Installation and Maintenance

The installation of native applications can depend on the application environment and the device brand, but the current trend for the discovery and delivery of native applications in various platform environments, such as on smart mobiles and smart TV platforms, is to acquire them from “app stores”. Applications are published to an app store (or market), where they are reviewed by the store managers. Once approved, applications can be downloaded and installed on the device when users request them. Updates also require the same review and approval process, and must be carried out specifically for each different platform.

Web applications on non-desktop devices are accessed in a variety of ways, depending on the target platform. The so-called “smart devices” (*i.e.*, smart phones and smart TV devices), as well as mobile phones, allow free searching for and access to any site available on the Internet. In contrast, some other interactive TV experiences only allow access to the Web applications included in standard marketplaces. However, hybrid solutions are also possible, such as the placement of Web applications (for both mobile and TV) inside native applications which are then sold through the common application stores. Web applications do not need to be reviewed and approved by the managers of any application marketplace, and they are updated simply by modifying the Web application on the server side. All the platforms then receive the updated version without modifications to the client system.

### 2.3. User Experience

Native applications are usually faster because they reside within the device platform itself and can take advantage of the system's UI functions. These functions also enable a set of visual effects that are shared by the rest of the system's applications. This results in a better user experience by facilitating the usability of applications on small screens.

Following the arrival of HTML5, Web applications can now be increasingly integrated within the system (especially on mobile devices). In addition, their structure can be semantic and more functional, and JavaScript functions can effectively meet the requirements of most applications. While some device functions (such as accelerometer, video and audio) are now supported in HTML5, other device-specific features are still under discussion. Two examples are the access to phone services (e.g., contacts, agendas, *etc.*) and the management of device-associated interfaces (e.g., webcams, microphones, USB devices, *etc.*) through the <device> tag. This is enough for most applications, but the rest still need it to be natively implemented, especially for non-mobile devices. HTML5 is still evolving and new features will be added, but it is currently not supported by many non-smart devices.

### 2.4. Development

The development of ubiquitous services as native applications requires specific knowledge of each particular platform, due to the varying underlying programming languages, SDKs, style guidelines and developmental infrastructures. This implies that there are usually higher development costs because of the need for a greater number of developers with different skills, and the specialization of developmental tools and frameworks. For building Web applications, developers mainly need knowledge of HTML, CSS and JavaScript, thus avoiding the need to learn new programming languages in order to code native applications. This allows the reusability of the existing development teams and developmental infrastructure for any new target platform. However, any platform may require specific content adaptations for the supplied Web sites in order to achieve the optimum user experience.

### 2.5. Our Conclusion

While platform support and user experience may still be better for native applications, Web applications can offer a more attractive solution for application providers by facilitating (and reducing the costs of) the distribution, updating and development of ubiquitous applications. Moreover, the platform support and user experience for HTML5 is rapidly being improved, and users will become increasingly used to Web applications on non-desktop devices as new applications become available. Finally, we firmly believe that the ubiquity achieved by Web applications through the principle of “build once, run anywhere” is the key to implementing ambient intelligence user applications. This consideration supports the choice of HTML5 for building the subtitling system proposed in this paper.

## 3. Web-Based, Scalable and Customizable Subtitles for AmI

Taking advantage of the underlying ubiquity of the Web, we have designed and successfully implemented a video subtitling system that enables subtitle format customization and its adaptation to the needs of the user/application. It is based on HTML5 and the new features which enable SVG and SMIL. Since the standardization of the HTML5 video component and its acceptance by the major Web browsers, there has been some implementation of the synchronization and presentation of subtitles attached to a <video> tag [8]. The majority of these changes make intensive use of JavaScript through third party libraries, which can imply a penalty with regards to the resulting performance and portability of the application. A different approach can be achieved by taking advantage of SMIL time properties for the synchronization of multimedia content. Instead of managing the content timeline programmatically through JavaScript, a web programmer can make use of the event management and timeline running on the SMIL engine of a Web browser. This can dramatically simplify the development of a time-based application such as subtitling by delegating the time management (*i.e.*, the presentation time of each subtitle or caption) to the browser, instead of this being managed by JavaScript.

### 3.1. Related Work

Jan Gerber was one of the first to develop a subtitle synchronization system for an HTML5 <video> tag, which resulted in the JavaScript library jquery.srt.js [9]. This library parses the HTML document, looking for subtitle containers which have been previously defined with the “srt” class, and attempts to load the related subtitles. Subtitles can be written directly in .SRT format into the subtitle container or accessed remotely through the address defined as a custom parameter into the HTML container. This implementation works correctly but relies on custom HTML attributes.

Adopting Gerber's approach, Dale proposed an evolution that demonstrates the benefits of including some HTML5 elements as child nodes of the HTML5 <video> container to associate a video component with time-aligned text [10]. This eliminates the custom attributes of the previous approach, but still relies on the same JavaScript library as subtitle synchronization for time-related issues.

Le Hegaret adapted Gerber's JavaScript library to demonstrate the viability of using Gerber's proposal with a different format for the presentation of time-based text in his HTML5 DFXP Player prototype [11]. He used the Distribution Format eXchange Profile (DFXP) of the Timed Text Authoring Format (TT AF) [12], which was created by the W3C for the conversion, exchanging and distributing timed text information amongst legacy distribution formats for subtitling and captioning.

Danilo proposed a completely different approach to multimedia synchronization within web-based environments [13]. This work presents an example of multimedia and graphic synchronization using SMIL to present SVG subtitles that are displayed over an SVG video. SMIL facilities allow the direct definition of time marks on subtitle text elements and specify the start time and duration of each subtitle. No JavaScript code is needed because the SMIL engine is responsible for maintaining the synchronization between video and subtitles.

### 3.2. Proposed Approach

**Figure 2.**
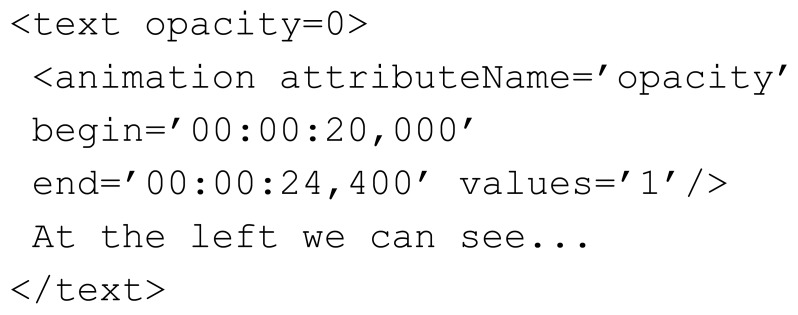
Example of an SVG text element with an SMIL animation for presenting a subtitle.

Inspired by Danilo's approach for synchronizing SVG subtitles through SMIL, this paper proposes a comprehensive solution for HTML5 video subtitling across multiple screens and devices. The solution enables the acquisition and parsing of remote subtitle files and allows dynamic modifications to the selected language and text format during video playback. It is robust against changes to the video timeline *i.e.*, the subtitles remain synchronized after functions such as pause, fast-forward, direct jump *etc.* are used. Subtitle text components are written in SVG in order to improve scalability and allow SMIL animations; JavaScript is used for parsing functions, client-server communications, the dynamic creation of subtitle text elements, and the event management of the <video> container. SMIL manages the functions related to time and decides the visibility of subtitle text according to the “begin” and “end” attributes defined by the SMIL <animation> element. These time markers correspond to the times defined in the related .SRT subtitle file. Figure 2 shows a subtitle definition using SMIL animations within HTML5 documents. Text opacity is initially set to 0 in order to obscure the text, and the <animation> element specifies when this opacity attribute must be altered to set the text to visible. The time format is the same as in the .SRT subtitle files (*i.e.*, hours:minutes:seconds:milliseconds), and therefore no calculations or JavaScript are required in the translation. The SMIL engine of the Web browser manages the rest of the process in order to keep subtitles synchronized with the video content, and so the application developer is relieved of this responsibility.

**Figure 3.**
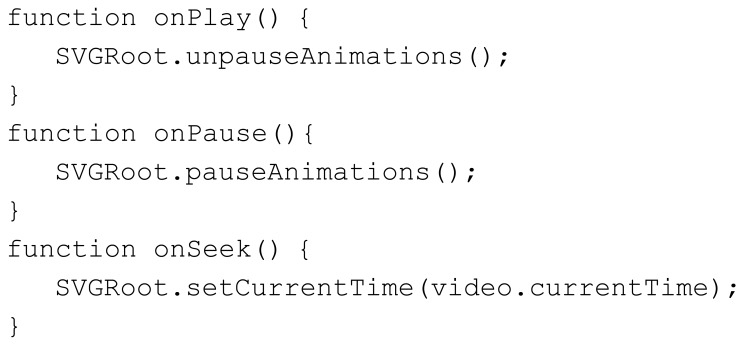
JS code of the video events added to the SMIL container for synchronizing subtitles.

One interesting novelty provided by HTML5 is the support for embedded SVG and SMIL code in the document in the same way as for any other HTML tag. These elements are also included as part of the Document Object Model (DOM) which means that they can be accessed and modified through JavaScript and CSS in the same way as any other HTML tag. By including SVG subtitles as part of the Web document, any web application can natively present customized subtitles, almost as in those platforms with the suitable web browser. However, this basic implementation is insufficient to support user-driven changes to the video timeline (e.g., pause, seek in time) because the timeline managed by the SMIL engine must also be notified about these changes. This is important for keeping the subtitles synchronized with the video content. When the user modifies the video playback timeline, any web application can automatically apply the suitable modifications to the SMIL timeline via the JavaScript API provided by HTML5 for the purpose of SMIL time-management. For basic playback management, three basic video properties are required: onplay, onpause and onseeking. Figure 3 shows an example of the JavaScript functions required to control the SMIL timeline. When the user pauses, plays or searches the video, the HTML5 <video> container elicits these JavaScript functions. The SVGRoot element of the example stands for the root element of the entire SVG document (*i.e.*, <svg>) and the variable video references the <video> container itself.

**Figure 4.**
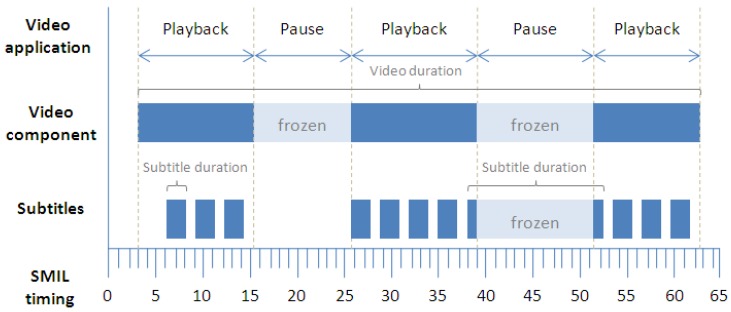
Strip diagram of basic timing support together with two video pause operations.

This behavior is represented in Figure 4, which illustrates the basic synchronization schema between the video component and its subtitles. Two video pause operations are also included in the diagram. When the user pauses the video component through the video player application provided, or the system elicits an event to pause the video (such as when the user switches between system applications), the JavaScript functions spread the pause event to the SMIL timeline. This helps to keep the subtitle timeline synchronized with the video according to the subtitle presentation times. If a subtitle element was being presented during the pause operation, it is frozen until the video playback is resumed. While the SMIL timeline is paused, all its time-dependent components also remain paused until the time is resumed. Subtitles are synchronously presented depending exclusively on the SMIL timeline and its own presentation time.

**Figure 5.**
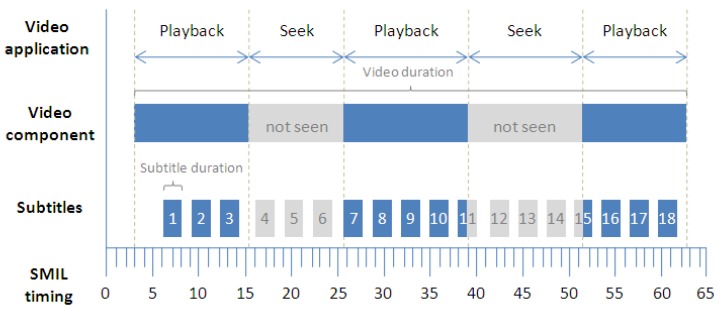
Strip diagram of basic timing support together with two video seek operations.

When the user generates jumps in the video timeline by seeking a concrete time position, the video application sends a signal to the SMIL timing container indicating that the time position must be updated. After the update, each subtitle component is presented according to its new current position on the SMIL timeline. Figure 5 outlines this behavior when the jump is forward in time.

**Figure 6.**
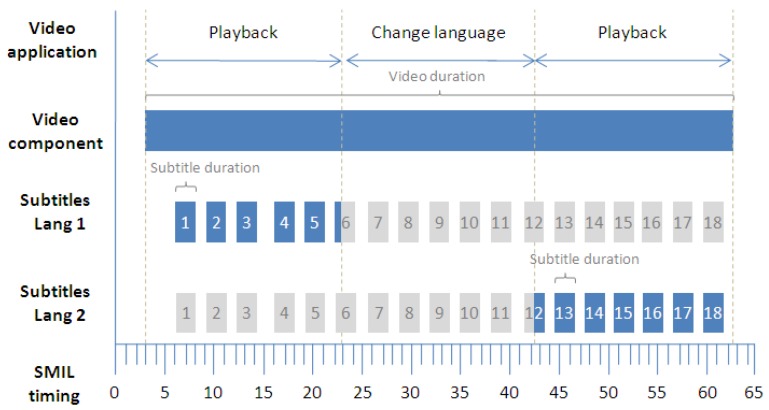
Strip diagram of basic timing support with a subtitle language change operation.

The operation to change the subtitle language (which is outlined in Figure 6) requires the loading of a new subtitle file (according to the SRT specification). This file can be obtained and loaded dynamically from a remote location while the video is playing. This loading operation can add a non-predictable latency, depending on the quality and speed of the connection required to obtain the new file.

Since the user generates the language change event until the system is able to present the new language subtitles (language 2), the subtitles of the current language (language 1) can be still displayed in order to maintain the subtitle continuity. However, the preliminary tests driven using the system indicate that this can be confusing for the user because somehow the system does not seem to immediately react to the user action. Instead, if the current language (language 1) disappears when the user begins the action, he or she gets the impression that something has happened and that the system is working. For system testing, we simulated latencies of up to 15 seconds, which is the default HTTP connection timeout for the Apache 2.0 web server. In practice, the subtitle file is usually obtained in a much shorter time, due to the file size (less than 100 KB for a 2 hour film) and suitable connection quality.

**Figure 7.**
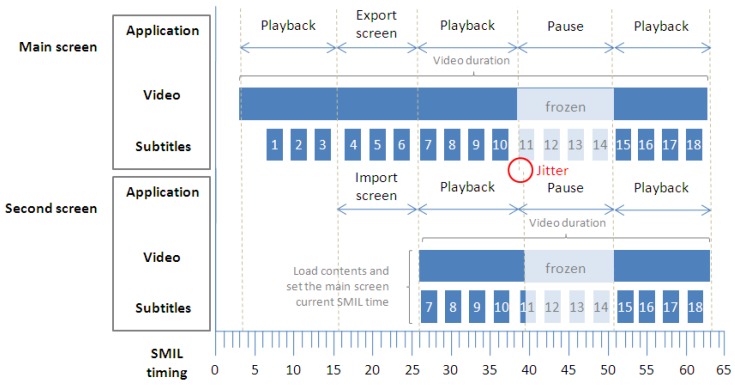
Strip diagram of basic timing support with a screen export operation for a second screen.

Finally, the screen export operation required for multi-screen scenarios is responsible for the synchronization between the main screen and the secondary screens. As shown below in Figure 7, when a secondary device is connected to the main system in order to obtain a complementary subtitle language, the system exports its SMIL time container onto the secondary device, taking into account the starting time and the current time. This means that the main system and the secondary device share the same time reference, maintaining the synchronism between both systems as long as the main system does not modify its own timeline. When this occurs, the main system sends a signal to the secondary device to update its own timeline according to the main system. In this case, it is assumed that both systems are operating within the same environment (e.g., the home environment), so the latency of this signal is negligible or short enough to ensure an acceptable user experience. However, if a pause operation generates a jitter between both paused systems, the secondary device may display the content slightly ahead of time (for instance the next subtitle may be displayed on the secondary device while the main system has yet to display it). When this occurs, the inconsistency is resolved at the time of the next playback operation, when the main system again sends its current SMIL time in order to resynchronize both systems. This particular point is included within the user experience evaluation conducted as part of this research, which is described in Section 5.3.

## 4. Analysis of the Current Platform Support

The platform support for the proposed solution depends on the availability of a web browser and its support for some HTML5 features, such as video and audio management, SVG and SMIL animations. Although a variety of browser plugins and SVG players exist, we have focused on browsers with native support for SVG in order to ensure maximum ubiquity. In the following section, we analyze the support for these features in three different application environments: desktop, mobile and TV.

### 4.1. Desktop Environments

Nowadays, all major modern desktop web browsers provide support for HTML5 and SVG to the level required by the solution proposed in this paper. An exception is Internet Explorer, which can present some problems with SMIL animations. Google's announcement in August 2010 that it had begun indexing SVG content (both standalone files as well as those embedded in HTML5 documents) and examples of successful applications such as that by Wikipedia (which presents SVG images) all add to the case for a wider acceptance of the format. Table 1 summarizes the support of the proposed subtitling solution provided in the most commonly-used desktop web browsers. It presents the main features which are required to build the proposed subtitling solution and their availability in the web browsers. As this analysis was also previously conducted in mid-2011 (see [14]), the table shows the evolution from those results (the left-hand column for each web browser) to the current state of support for the solution (the right-hand column). The table also shows that the development teams of major web browsers are extensively involved in providing full support to HTML5 and related technologies such as SVG and SMIL. This is more evident in the mobile side of the business, where the industry seems to have progressed more rapidly in this direction.

According to the table, the subtitling solution proposed in this paper is supported by all major desktop web browsers with the exception of Microsoft's Internet Explorer, which has not been updated in this regard from the previous version and still requires a third party library or plugin to deploy SMIL animations. Moreover, as far as we are aware, the support for this feature in the next version of the browser (10.0) will also not support this feature.

### 4.2. Mobile Environments

The mobile industry is rapidly adopting HTML5, which facilitates the development of ubiquitous applications for AmI. The support recently provided for new HTML5 features by all major mobile web browsers is proof of this. Notably, less than one year prior to this, it was said that mobile web browsers were still some way behind when compared to the development of HTML5 within desktop browsers [14]. However, nowadays the support that mobile web browsers can provide to HTML5 and especially to SVG and SMIL is full comparable to that of desktop environments. As shown in Table 2, all the HTML5 features required for the implementation of the proposed subtitling solution are now supported by all major mobile web browsers. This not only means that the solution is totally feasible for the mobile market but also that all kinds of multimedia and AmI applications with content synchronization, scalable graphics and SMIL animations within these graphics are now accessible by most smartphone or tablet users. This global support should lead to these kinds of applications becoming more common throughout the mobile application market. As with the evaluation of the browser support for desktop environments (Table 1), Table 2 presents the evaluation of the required HTML5 features in the analysis conducted in mid-2011 (left-hand column) compared to the current state of mobile web browsers (right-hand column).

### 4.3. TV Environments

The situation for interactive TV is even more incoherent than for mobiles because the technological details concerning what actually constitutes a “smart TV” have not yet been clearly defined. Currently, three main approaches coexist as smart TV platforms:

1. Device and Internet operators have provided their own solutions. In the case of Apple and Google (which have created AppleTV and GoogleTV respectively), the former provides an integrated Set-top Box (STB), whilst the latter provides a framework which can be deployed on prepared STBs and TVs.
2. TV manufacturers build Internet-capable devices that combine the TV video flow with web applications and widgets. Their browser implementations provide little support for HTML5 features as they have very limited resources (even less than mobile devices).
3. Some Internet video service providers (e.g., Netflix and Boxee) are interested in collaborating with TV and STB manufacturers to deploy their services on a variety of terminals. In any case, the support for HTML5 and SVG animations on smart TV devices depends on the device resources available to deploy a web browser that supports the features required. Fortunately, the Open IPTV Forum recommends the use of SVG (SVG Tiny 1.2) for declarative environment applications on the client side [15] and some initiatives for the standardization of hybrid digital TV platforms such as the Hybrid Broadcast Broadband TV (HbbTV) [16] follow this model. Even so, and according to Ericsson, the SVG specification could benefit from some extra features in order to make SVG more suitable for TV (for example, support for TV-related remote control keys, the ability to seek through programs and speed for fast forwarding/rewinding, *etc.*).

## 5. Three Different Application Use Cases

The proposed approach for video subtitling in AmI environments has been validated through its implementation in three different examples in order to demonstrate its usefulness and ubiquity. The first extends the functionality of an HTML5 <video> container for web applications onto desktop browsers; the second offers a solution for video subtitling within web-based interactive TV environments; and the third makes use of mobile devices to present personalized subtitles on a TV's second screen.

### 5.1. The SVG Subtitle Access Bar

The SVG Subtitle Access Bar (SVG-SAB) is a web widget which extends the functionality of any HTML5 <video> component to enable advanced subtitle functions. It facilitates access to video content within Web applications by adapting subtitles to the needs and preferences of the user. This widget applies the solution proposed in this paper to synchronizing subtitles and video content on the client side using SMIL animations. This keeps content synchronized even if the user dynamically changes the language, the subtitle text format and the position of the text on the screen. Although it can be implemented in any web application, the main goal of the SVG Subtitle Access Bar is experimenting with video subtitles for research purposes [17–19]. It enables the study of the optimal subtitle format according to the context requirements and the user needs. All features can be altered dynamically via a web interface, which means that the widget can be controlled by a third-party application managing the user and environmental variables. This management may enable the dynamic adaptation of the subtitle text color (depending on the contrast with the video content and the global environmental lighting), the automatic change of language according to the browser-defined language, or the splitting of both sources (video and subtitles) for the separate presentation of one or both of them on a second screen. As both sources are merged on the client side, unlike traditional subtitled video content, they can work separately but synchronously in the same application environment. Besides the customization of the subtitle format, this also enables the positioning of subtitles beyond the video window and even beyond the main screen.

**Figure 8.**
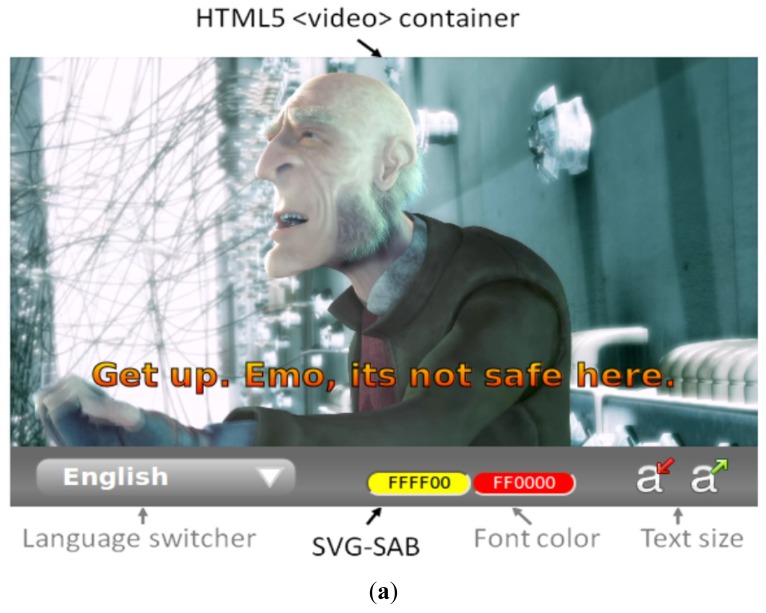

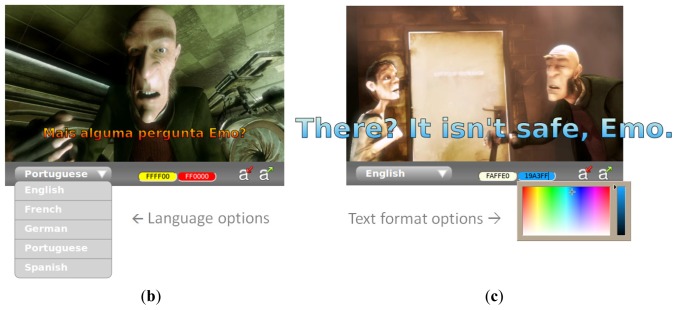
The SVG Subtitle Access Bar presented on a desktop browser. It is attached to an HTML5 video component for synchronizing the subtitles with the video content. (**a**) presents the main features of the described widget. (**b**) is an example of the dynamic language switching function. Different languages can be shown during the video playback without synchronization failures. (**c**) is an example of the dynamic subtitle customization. The text color and gradient can be changed using a built-in color palette and the text can be manually resized. Size can be extended beyond the video window if necessary.

Figure 8 shows a snapshot of the SVG-BAR attached to a video component playing a subtitled video. Subtitle languages can be selected and switched dynamically from remote .SRT files. Each subtitle is added as a DOM node with its corresponding presentation time, and SMIL manages these times in order to display each piece of text on the screen. Developers do not need to take into account either the current playback time or the subtitle sequence order to maintain the content synchronization.

When a language switch is applied, the widget removes the previous subtitles and adds the new ones without a delay in synchronization because the SMIL timeline is responsible for presenting each subtitle at the correct time. The SVG-SAB widget also enables modifications to the subtitle text color and size in order to enable its adaptation to the requirements of the video, application, user and context e.g., text color can be adapted to achieve a better contrast, depending on the lighting content and context. Adaptation of the subtitle may also be necessary in order to overcome user difficulties in accessing content. The use and functionality of these subtitles is multiple: for language teaching and language therapy [3], for people with visual impairments or color blindness, for the elderly and people who speak other languages, or for those with literacy or comprehension difficulties [17,18].

The content used for the experimentation conducted as part of this research, as well as the film shown in the screenshots presented in this paper, is “Elephants Dream” (2006) [20], an open animation movie initiated by Blender Foundation to promote their free software application for animation.

### 5.2. The Positioning of Subtitles on Interactive TV

On TV, subtitle customization enables better access to video content, especially within the environment of interactive TV. Subtitles can be properly positioned and sized to ensure maximum readability, while the video content continues playing through an interactive user interface. Subtitle position can also be modified in order to position it wherever is most comfortable for the user [20]. This feature has been applied (as in Figure 4) to constructing an interactive application for TV that presents video subtitles which are always readable even if the video window is reduced in order to display the rest of the interactive content. Due to the time management of the SMIL browser engine, content is always synchronized, even if it is visually separated on the screen.

**Figure 9.**
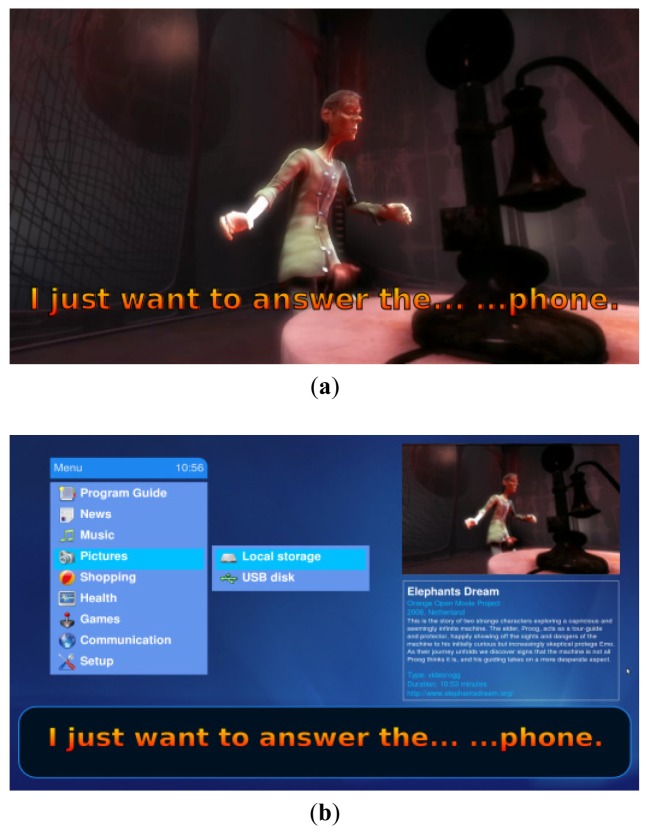
Two screenshots showing the positioning of subtitles within an interactive TV environment. In (**a**), the user is watching subtitled video content on the TV. He then accesses the interactive element and the video playback is reduced to a corner of the screen as shown in (**b**). Here, subtitles are still presented at the normal size and positioned in the same place as before.

A proper positioning of subtitles can also be automatically generated by the video component when it is resized to be accommodated by an interactive application. As the enhanced video component is responsible for managing and presenting the subtitles, the interactive application does not need to know the precise details of the subtitles being presented beyond accommodating them in order to ensure a correct visualization while the user is browsing the application. Figure 9 shows an example of an interactive application for TV which implements this smart widget for presenting video subtitles on connected TVs. The video is being played on a TV and the subtitles are presented in the language selected by the user. When the user accesses the interactive application, the video component is resized and accommodated within the interactive interface; the widget then automatically detects that it has been resized and presents the subtitles accordingly. This is a very simple function but outlines the capabilities which can be achieved by the proposed solution when presenting subtitles in smart environments. These environments, surrounded by sensors and smart applications for detecting the needs of the user, are only feasible if their components collaborate through common APIs which facilitate their integration within the whole system. This is the case for web components and applications, which are based on common standards, unlike the native applications written for specific platforms.

### 5.3. Presenting Subtitles on a Second Screen

Within the home environment, a mobile device can be used as the TV platform's second screen [21] on which to extend its content and management options. Mobile devices can become an advanced remote controller, as well as the support on which to present either the same content as on the main TV set or one or more complementary pieces of content. Access to video content can benefit from this feature as it allows each user personalized access to video subtitles through their own smart mobile phone. This can facilitate access to content for people with special requirements (e.g., the elderly, children and disabled people), and thus improve the family balance. Outside of the home environment, this solution can be applied in public spaces such as theaters, museums and conferences in order to provide subtitles or complementary information to all users regardless of the language they speak. This complementary information can be permanently synchronized with the main content and enhances the accessibility not only of specific content but of the whole smart environment. In public smart spaces, a multi-screen solution like this can facilitate cultural access for people with special requirements. In theaters, for instance, a film or a play can be followed by people who do not understand the spoken language by accessing the relevant subtitles via the theater's own mobile smart device, regardless of the device operating system. Here, a mention has to be made regarding the production of these subtitles for live events or plays, where some element of improvisation is to be expected. Although in these cases the subtitle production is not usually an easy task and we do take this into account, this is beyond the scope of this research. Our proposal asserts that while subtitles are produced they can be sent to multiple (and different) devices without synchronization problems.

We have constructed a web-based mobile application to present video subtitles which are synchronized with TV content, extending the TV screen onto a mobile device. In a home environment, this mobile application connects to the media center, which serves the interactive TV application with the synchronized video subtitles (as shown in the previous section), via the WLAN network. The user receives the subtitles of the current video content on his or her mobile device and can change the language, size and color of subtitle texts independently from the TV stream or of any other connected device. This mobile web application applies the same SMIL subtitle synchronization principle proposed in this paper. The main difference with regards to implementation from the previously highlighted examples of the use of the application is the synchronization requirements between the media center and the user device which is acting as a second screen.

**Figure 10.**
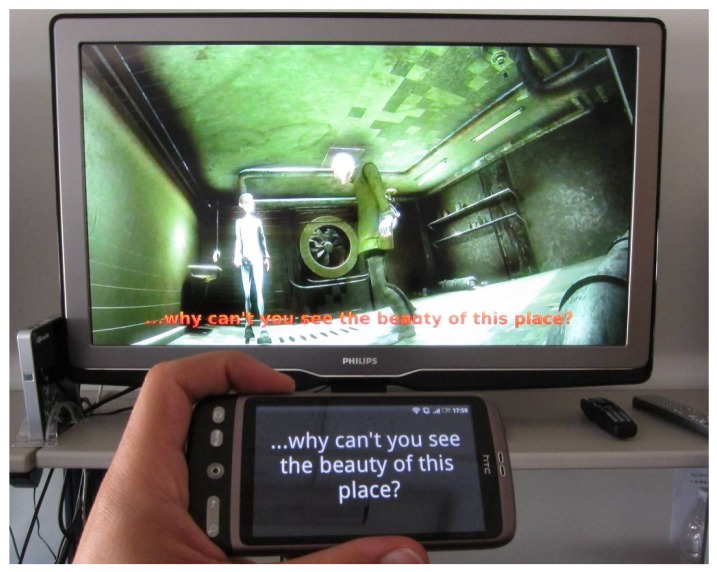
An example of a second screen application for receiving subtitles on an Android 2.2 platform.

In our implementation, the user device application receives the current playback time from the media center, which acts as a server. The Media Center and device SMIL timelines are then synchronized and the mobile browser engine can then present each subtitle at the correct time. This enables the extension of multimedia content onto all connected devices available in the user environment and can be integrated in AmI systems via its communication with the sensors and applications, which makes possible the automatic recognition of the user's needs. Figure 10 shows an example of the use of this functionality for accessing TV content in order to receive subtitles from an Android 2.2 platform as complementary information. If the environment can detect the position of the user, the system can send subtitles (or indeed the whole movie) to those connected users who may have left the room. By detecting the ambient noise, the system can also send subtitles to users if it is difficult to hear the audio content.

## 6. Evaluation of the User Experience

The user experience provided by the proposed solution for subtitle synchronization across multiple screens and different devices in AmI environments has been evaluated in order to validate its acceptability by end-users. The evaluation scenario is similar to that presented in Section 5.3, where users watching TV content can receive complementary content on their smartphones. In the case of the evaluation, each participant was asked to watch a short movie whilst reading subtitles on their own smartphone in order to allow them understand the storyline of the fragment presented.

The first and second scenarios presented herein have not been evaluated by users because these examples do not present remarkable issues, the exception being a possible lack of synchronization between video and subtitles which is also evaluated in the case of the third scenario. Moreover, the techniques applied in the first two scenarios can be enhanced in the medium term by the HTML5 standard and the suitable definition of subtitles and captions within web documents. In contrast, the solution applied in the third scenario is a multiplatform approach for second screens which is out of the current scope of the standard definition tasks.

The main objective of this experiment was the validation of three aspects: (1) the platform independence of the proposed solution—letting the subjects use their own smartphones for the experiment. Although this could provide unpredictable results due to the loss of control over the variables which may affect the user platform (such as device manufacturer, operating system, selected web browser and any other installed function which may affect the running of the test), the experiment with users' own smartphones provided a real test of what is expected of a service for use in public spaces. A secondary reason for taking this decision was the familiarity which smartphone users have with their own devices, avoiding possible usability issues which may be experienced by users without previous training on this kind of platform; (2) the viability of smartphones as secondary screens based on the proposed solution and its acceptability for use at home or in theaters; (3) the quality of synchronization between the main content (in this case, the content displayed on the TV) and the secondary devices accessing complementary information (in this case, smartphones accessing subtitles).

### 6.1. Evaluation Methodology

In order to drive this subjective evaluation, participants were recruited as volunteers to act as subjects for the experiment. A total of 15 subjects were selected, seven female and eight male, aged between 24 and 53 years old. The number of users chosen follows the samples used in similar subtitling experiments (see [2,3,17,18,20]), along with the methodology. A basic questionnaire was presented to participants in order to characterize the sample of subjects and to obtain information concerning their individual habits with regards to audiovisual content, their visual acuity (a basic test simply to check that they could understand the visual content at the viewing distance) and their degree of prior knowledge of the use of smartphones. Before this preliminary questionnaire, subjects were asked to bring their own smartphone to the experiment. Almost all the participants (86.6%) responded that they consume audiovisual content (*i.e.*, any kind of video content) every day via either a TV, a mobile device or a desktop computer. They all use their smartphone daily to access one or more Internet services (e.g., email, news, social networks, browsing the web *etc.*) and the majority (73.3%) sometimes does this whilst watching TV at home. Some of participants (46.6%) regularly consume subtitled audiovisual content, but this percentage rose (80.0%) when the subjects were asked whether they would consume more subtitled films if they were readily available. The smartphones they used were based variously on the Android (60%), iPhone (33.3%) and Windows Phone (6.6%).

For the experiment, subjects were asked to watch an open animation movie of 15 minutes duration called “Sintel” (2010) [22], which (as with the content used in the main part of this research) was also initiated by Blender Foundation. The movie was presented with sound disabled in order to force the participants to read the subtitles on their smartphones. The subjects' tasks consisted of: (1) using the smartphone to connect to the local network and access the application for subtitles; (2) freely watching the movie and reading the subtitles on the smartphone in order to understand the storyline; (3) changing the default language (Russian) to that of their mother tongue when first subtitle appeared; (4) answering a questionnaire regarding their satisfaction and the quality of the service. The questions presented were based on a 5 grade Likert scale [23] to obtain answers ranging from “Strongly disagree” to “Strongly agree”.

### 6.2. Evaluation Results

**Figure 11.**
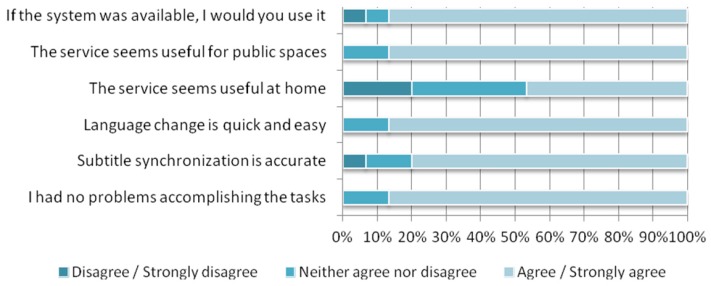
The summarized results of the user experience evaluation.

The results of the user experience evaluation (shown in Figure 11) are divided into 3 questions and answers relating to quality and 3 relating to acceptability and user satisfaction. The chart shows that the mobile web application used by subjects to access subtitles was simple enough to accomplish the required tasks. The connection and access to the application was easy and there were no remarkable issues with regards to the application usage. In addition, the operation to change the language was quick and easy for most of the subjects (and smartphones) involved in the study.

The acceptability of using a mobile device for accessing subtitles at home has been differently rated. On the one hand, although for certain situations it was seen as a useful function, some subjects did not feel that there was a need for this service at home under standard conditions. Using more devices to access complementary content must be *required* by the context, because it can be seen as unnecessary and obtrusive when not needed. This is usually the case for subtitles, which are currently presented on TV instead of on a mobile device. On the other hand, the level of acceptance of the service was totally different when applied to public spaces such as theaters, museums and the street. It seems that there are more situations in public spaces where this service is perceived as being useful or necessary. Finally, subjects largely agreed that they would use this subtitling service if it were available, particularly in public places.

Some further comments were related to improving the menu for changing the language in order to enhance visibility. Regarding subtitle synchronization, some subjects detected issues on their smartphones when changing language because their mobile browser spent a few seconds importing the new language file, which requires some DOM management. This function can be specifically optimized to run properly on devices with low resources in order to avoid these synchronization issues.

This approach has been demonstrated as being practical and has been properly adapted across different devices. The evaluation conducted provides information concerning the context where this service is perceived as being useful and may focus the attention of the service and application developers. Beyond the subtitling service, the acceptability of smartphones as multi-screen devices has not been questioned.

## 7. Conclusions

This paper presents a novel approach for video subtitling within AmI environments which enables multi-screen capabilities to provide timed complementary content to users anywhere and at any time. The benefits of building ubiquitous web-based applications have been compared with the native support offered by the main access platforms. The rapid evolution in the level of support for the new HTML5 capabilities by major web browsers is one of the key reasons for choosing this flexible and adaptable set of web-based technologies. This paper has focused on the use of some of these standards (HTML5, SVG and SMIL) to improve the accessibility to video subtitles within AmI environments by allowing their customization and adaptation to the user, device and context. The three cases presented provide examples of the application of the proposed approach for different user needs (e.g., language), on different devices (desktop, mobile and TV) and in different contexts (*i.e.*, at home and in public places). Moreover, the multi-screen capabilities have been validated though a subjective test of the user experience of a service generated for acquiring subtitles on the user's smartphone. The experiment has demonstrated the viability and acceptability of the proposed solution as well as the preference of the subjects surveyed for using the system in public places rather than at home. This should be taken into account when further research is conducted into this area.

## Figures and Tables

**Table 1.** Support of the major desktop web browsers for the proposed solution. For each browser, the left-hand column indicates the support provided by the available browser version at the time of our previous study (in mid-2011) and the right-hand column indicates the support provided by the current version of the browser (in early 2012).

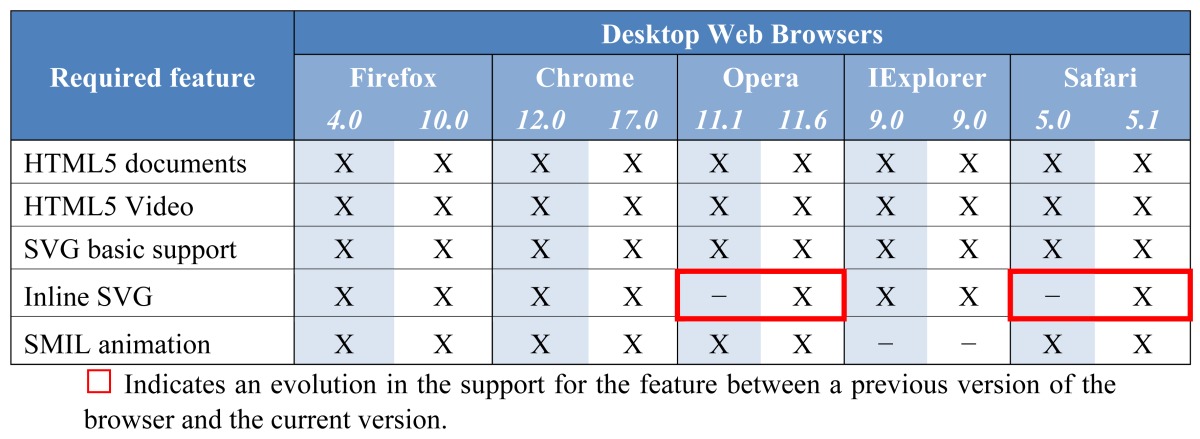

**Table 2.** Support of the major mobile web browsers for the proposed solution in. For each browser, the left-hand column indicates the support of the browser version available during our previous study (conducted in mid-2011) and the right-hand column indicates the support provided by the current browser version (in early 2012).